# Cardiomiopatia Hipertrófica – Revisão

**DOI:** 10.36660/abc.20190802

**Published:** 2020-11-01

**Authors:** Silméia Garcia Zanati Bazan, Gilberto Ornellas de Oliveira, Caroline Ferreira da Silva Mazeto Pupo da Silveira, Fabrício Moreira Reis, Karina Nogueira Dias Secco Malagutte, Lucas Santos Nielsen Tinasi, Rodrigo Bazan, João Carlos Hueb, Katashi Okoshi

**Affiliations:** 1 Universidade Estadual Paulista Júlio de Mesquita Filho Faculdade de Medicina de Botucatu BotucatuSP Brasil Universidade Estadual Paulista Júlio de Mesquita Filho – Faculdade de Medicina de Botucatu - UNESP, Botucatu, SP - Brasil

**Keywords:** Cardiomiopatia Hipertrófica/genética, Morte Súbita, Insuficiência Cardíaca, Ecocardiografia/métodos, Hipertrofia Ventricular

## Abstract

A cardiomiopatia hipertrófica (CMH) é a doença cardíaca de origem genética mais comum, cuja principal característica consiste na hipertrofia ventricular esquerda que acontece na ausência de outras patologias que desencadeiam tal alteração. A CMH pode se apresentar desde formas assintomáticas até manifestações de morte cardíaca súbita e de insuficiência cardíaca refratária. Métodos de imagem contemporâneos de alta resolução e escores clínicos mais acurados vêm sendo utilizados e desenvolvidos no sentido de propiciar uma avaliação prognóstica e funcional mais adequada, bem como possibilitar a estratificação dos casos de maior gravidade. Nesta revisão, serão abordados esses aspectos, entre outros tópicos clássicos inerentes ao estudo dessa doença.

## Introdução

**Figura 1:**
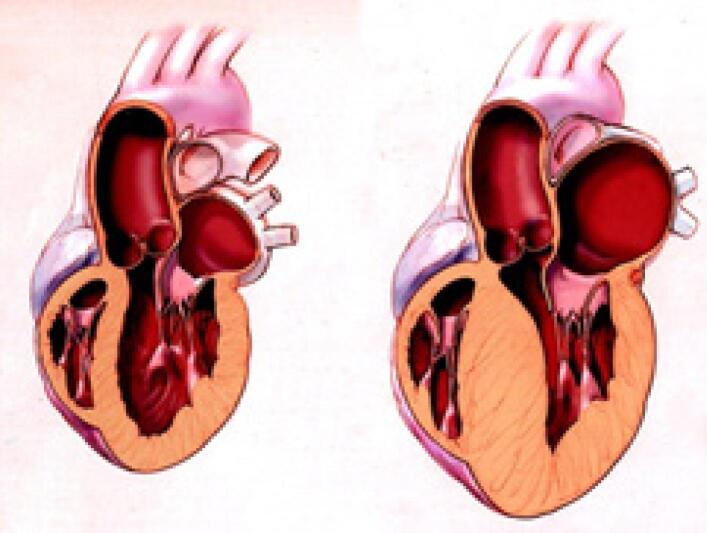
Esquemas de um coração normal (painel esquerdo) e um coração com CMH (painel direito).

A cardiomiopatia hipertrófica (CMH) é uma doença de causa geneticamente determinada que acarreta alterações estruturais na conformação cardíaca (Figura 1). A principal característica anatômica dessa doença é a hipertrofia ventricular esquerda (HVE) com várias morfologias na ausência de outras condições que justifiquem esse achado.^1^

A prevalência de CMH é relativamente frequente, estimada em 0,2% da população adulta.^2^ As suas manifestações clínicas são extremamente variadas, desde formas assintomáticas até insuficiência cardíaca (IC) avançada, dentre outras apresentações que culminam em morte súbita.^3^

Por outro lado, avanços no tratamento da CMH resultaram em uma taxa de mortalidade anual que atualmente é inferior a 1%.^4^^,^^5^

Desse modo, é um assunto de grande interesse devido a sua prevalência significativa e à importância da identificação precoce dos grupos em risco, como os atletas.

### Bases genéticas

As análises genéticas da CMH identificaram uma série de mutações em mais de 11 genes que codificam proteínas sarcoméricas.^6^ A CMH pode ocorrer em um padrão de herança autossômica dominante com expressividade e penetrância variáveis relacionadas à idade ou como uma nova mutação em casos sem relação com a família.^7^^,^^8^ A mutação predominante é a mutação missense, na qual um ácido nucleico é substituído por outro, com alteração posterior do aminoácido traduzido e da propriedade funcional da proteína resultante. Inserções e supressões também são mutações comuns envolvidas na patogênese da CMH, as quais desencadeiam a produção de proteínas anormais.^8^

Os pacientes com CMH apresentam algum tipo de alteração genética em aproximadamente metade dos casos.^9^^,^^10^

**Figura 2:**
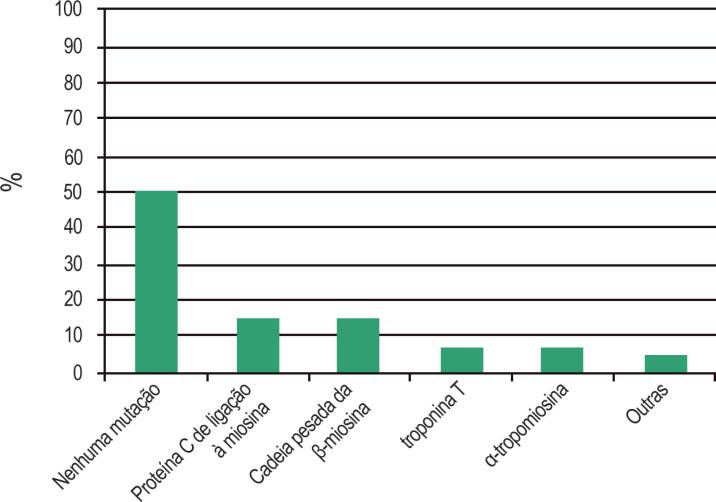
Distribuição de mutação dos genes na CMH (Adaptado de Maron BJ et al.^8^)

A maioria das mutações afeta os genes que codificam proteínas contráteis do sarcômero cardíaco: troponina T e cadeia leve de miosina I, cadeia pesada de miosina alfa e beta, proteína C de ligação à miosina, α-actina, α-tropomiosina e titina. Entretanto, mutações nos genes codificadores de proteínas não sarcoméricas também já foram identificadas em pacientes com CMH.^11^ Os genes mais comumente relacionados ao desenvolvimento de doenças são a cadeia pesada da β-miosina (MYH7), a proteína C de ligação à miosina (MYBPC3), e a troponina T (TNNT2)^12^ (Figura 2).

A patogenicidade de uma mutação é avaliada probabilisticamente utilizando-se uma série de critérios que determinarão o risco de desenvolvimento da CMH.^13^

Também é importante destacar o conceito de fenocópias no contexto da CMH. Esses pacientes têm o fenótipo da CMH sem as mutações genéticas da CMH, mas em vez disso apresentam outra doença que resulta em algum problema cardíaco similar, tais como Doença de Fabry, cardiomiopatia LAMP2, PRKAG2 e/ou amiloidose.

### Achados patológicos

**Figura 3:**
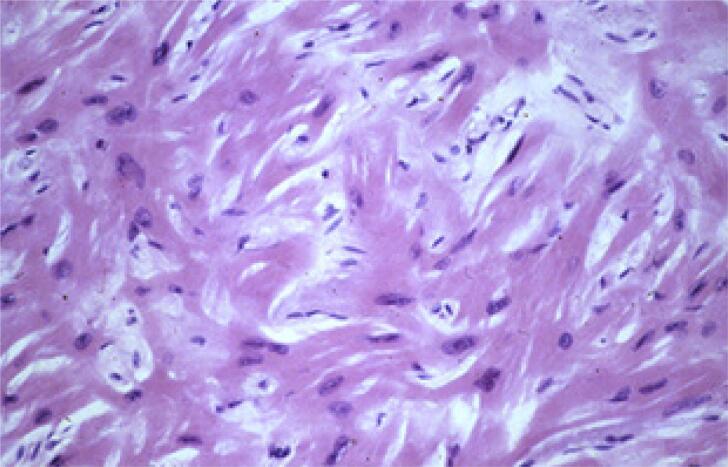
Desarranjo de miócitos do tecido miocárdico de um paciente com CMH.

Na CMH, o exame histopatológico mostra fibras miocárdicas hipertrofiadas distribuídas de forma desorganizada e interpostas em uma quantidade variável de fibrose intersticial^14^ (Figura 3).

Além disso, as arteríolas coronárias intramurais são estruturalmente anormais e apresentam diminuição da área intraluminal com deterioração da capacidade vasodilatadora, o que ocasiona um fluxo sanguíneo ineficiente ao estresse.^15^ Com o passar do tempo, repetidos episódios de isquemia levam à morte celular, e a correção é mediada pela substituição por tecido fibrótico.^14^

Diferentes tipos de apresentações anatômicas da CMH já foram relatados. O tipo mais comum é a hipertrofia septal assimétrica (presente em >75% dos casos), seguida das apresentações apical, concêntrica, medioventricular e lateral.^16^

### Fisiopatologia

Os sintomas da CMH estão relacionados à combinação de disfunção diastólica, obstrução da via de saída do ventrículo esquerdo (VSVE), regurgitação mitral, isquemia miocárdica e arritmias. O fator mais comum que contribui para o desenvolvimento da obstrução da VSVE é o movimento anterior sistólico da valva mitral (MAS) contra o septo interventricular (SIV). O MAS ocorre devido à alta velocidade do fluxo sanguíneo através da VSVE que arrasta a cúspide anterior da válvula mitral para o septo interventricular, resultando em um obstáculo direto à passagem do fluxo sanguíneo através da via de saída.^17^

Além disso, a combinação de desarranjo de miócitos, desordem autonômica, HVE, isquemia e fibrose miocárdica produz um substrato arritmogênico suficiente para o desenvolvimento das principais arritmias observadas em pacientes com CMH.^2^

Essas características não aparecem simultaneamente, e uma classificação de 4 estágios foi proposta para auxiliar o diagnóstico e o manejo de pacientes: CMH não-hipertrófica, fenótipo clássico, remodelação adversa e disfunção evidente.^18^ Conforme o paciente avança através dos estágios, ele sofre uma perda de fração de ejeção, um aumento da massa ventricular esquerda, uma piora da disfunção microvascular e diastólica, uma intensificação dos sintomas e uma perda de obstrução prévia da via de saída do ventrículo esquerdo, que geralmente se inicia no estágio 2.

### Apresentações clínicas

Os sintomas associados à CMH estão relacionados com os perfis da doença, incluindo a apresentação assintomática, morte cardíaca súbita/arritmias ventriculares, obstrução, insuficiência cardíaca com fração de ejeção preservada, fibrilação atrial/acidente vascular cerebral (AVC) e insuficiência cardíaca com fração de ejeção reduzida. Embora muitos pacientes com CMH não apresentem sintomas ou apenas tenham sintomas menores, outros podem apresentar dispneia ao esforço, fadiga, dor no peito, pré-síncope e síncope, durante ou logo após o esforço, e palpitações.^19^

Já existe uma correlação bem estabelecida entre a presença ou magnitude da obstrução da VSVE e a presença de sintomas.^20^

Para a maioria dos pacientes com CMH, a HVE não é progressiva e é compatível com uma longevidade normal, com uma taxa de mortalidade anual de cerca de 1%.^21^

Por outro lado, um grupo pequeno de pacientes apresenta o risco de desenvolver sintomas relacionados à progressão de insuficiência cardíaca sistólica, morte súbita, e fibrilação atrial relacionada a fenômenos tromboembólicos.^22^

A presença de um gradiente de pressão na VSVE em repouso ou provocada por exercício ocorre na maioria dos pacientes com CMH.^23^ A obstrução significativa em repouso é um fator independente para um pior prognóstico e progressão à insuficiência cardíaca.^24^

O exame físico de pacientes com CMH pode revelar achados normais a presença de vários sinais, tais como quarta bulha (B4), sopro sistólico de regurgitação na borda esternal esquerda inferior, desdobramento paradoxal da segunda bulha cardíaca (B2), impulso apical aumentado, e frêmito sistólico. Adicionalmente, os pacientes com obstrução da VSVE podem apresentar um sopro sistólico de ejeção na borda esternal esquerda que geralmente se irradia para a borda esternal superior direita e que pode aumentar ao se levantarem da posição de cócoras e na manobra de Valsalva.

Pode ser constatado pulso arterial *bisferiens* e presença de pico sistólico em forma de cúpula, enquanto uma onda “a” proeminente é detectada no pulso venoso.

### Exames complementares

**Figura 4:**
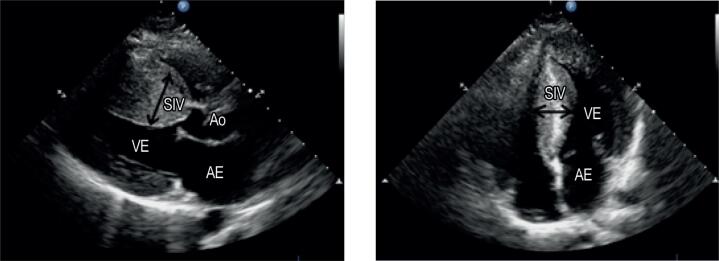
Ecorcardiograma transtorácico revelando hipertrofia assimétrica do septo interventricular. SIV: septo interventricular; VE: ventrículo esquerdo; AE: átrio esquerdo; Ao: raiz aórtica. (Serviço de Ecocardiografia do HC - Faculdade de Medicina de Botucatu - UNESP).

– Eletrocardiograma (ECG): Esse teste deve ser realizado em todos os pacientes com suspeita de CMH. Um ECG normal é atípico, visto que ele ocorre em menos de 10% dos pacientes com CMH, e é muito sensível na identificação da doença.^25^ Esse grupo de pacientes tende a apresentar um prognóstico melhor em relação àqueles que apresentaram alterações eletrocardiográficas.^26^ O padrão anormal mais comum é a presença de alterações localizadas ou difusas na repolarização ventricular. Outros achados podem incluir sinais de hipertrofia ventricular esquerda, inversão da onda T nas derivações esquerdas, e aumento do átrio esquerdo. Ondas “Q” profundas e estreitas podem ocorrer em V5 e V6.
– Ecocardiograma: O ecocardiograma é um exame essencial tanto para a confirmação do diagnóstio quanto para as avaliações evolutiva, funcional e prognóstica.^27^ O ecocardiograma transtorácico pode mostrar a morfologia do coração, estimar a função sistólica e diastólica, avaliar a presença e a gravidade do gradiente na VSVE, bem como determinar o grau de regurgitação mitral. Os principais achados ecocardiográficos associados à CMH são HVE (principalmente se for assimétrica e envolver a parede ântero-lateral ou o septo), aumento do gradiente na VSVE, e o movimento anterior sistólico da valva mitral (Figura 4).

Os pacientes que permanecem sintomáticos e não apresentam obstrução em repouso podem ser submetidos à ecocardiografia de esforço para induzir um gradiente e, posteriormente, ajustar o manejo e o tratamento de acordo com o resultado.^23^

**Figura 5:**
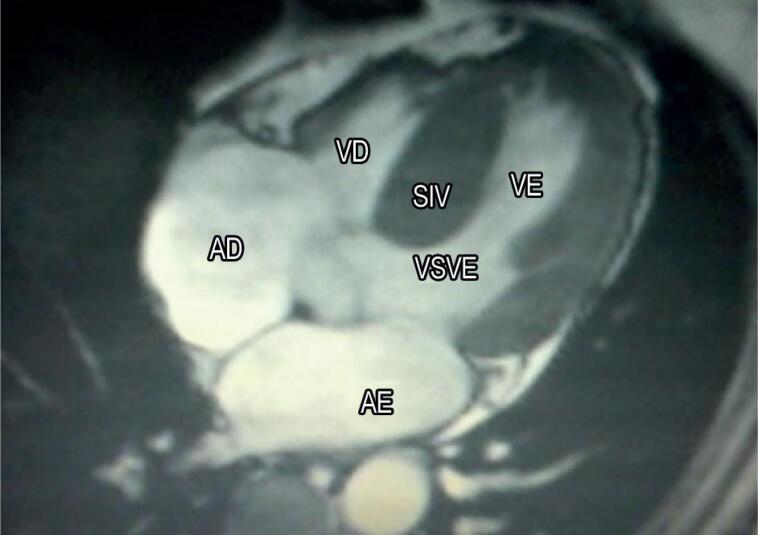
RMC de um paciente com CMH e apresentação septal assimétrica não obstrutiva. AE: átrio esquerdo; AD: átrio direito; SIV: septo intraventricular; VE: ventrículo esquerdo; VD: ventrículo direito; VSVE: via de saída do ventrículo esquerdo (Cortesia do Departamento de Radiologia do HC - Faculdade de Medicina de Botucatu - UNESP).

– ECG Holter: Esse exame é realizado como parte da estratificação do risco de desenvolver arritmias ventriculares e morte súbita, bem como para investigar palpitações e em pacientes com suspeita de fibrilação atrial.
– Teste de Esforço: Esse teste é tipicamente utilizado para estratificação de risco por meio da resposta da pressão arterial ao exercício e para investigar isquemia e arritmias.
– Ressonância Magnética Cardíaca (RMC): A RMC fornece imagens de alta resolução para avaliar as estruturas cardíacas. Além de conseguir identificar a hipertrofia em segmentos que não são exibidos na ecocardiografia, ela também mostra áreas de fibrose miocárdica, que geralmente são detectadas através do realce tardio de gadolínio, e são um dos fatores de risco para morte súbita, permitindo uma melhor caracterização das anomalias estruturais no aparato da válvula mitral^28^^–^^30^ (Figura 5).

### Tratamento

O início do tratamento se dá com medidas preventivas, tais como evitar a depleção do volume intravascular e restringir a prática de exercício físico intenso, com a recomendação individualizada do nível de atividade física para cada paciente.^31^^,^^32^ Outras medidas incluem a manutenção de drogas inotrópicas negativas, evitar o uso de vasodilatadores e a adoção de um tratamento apropriado para taquiarritmias.

#### Terapia medicamentosa

A terapia farmacológica é o tratamento de primeira linha para pacientes com sintomas de IC relacionada à obstrução da VSVE.^26^

O uso de medicamentos não é recomendado antes do desenvolvimento dos sintomas, já que não há evidências de que a terapia farmacológica mude a história natural de pacientes assintomáticos.

A primeira linha de tratamento inclui betabloqueadores. Atualmente, experimentos clínicos não apontam preferência por um betabloqueador específico, uma vez que eles não foram comparados. Entretanto, estudos relataram os benefícios de propranolol e sotalol, apesar de este último ser um agente antiarritmíco Classe 3, na redução dos sintomas e na diminuição de arritmias.

Caso os betabloqueadores não aliviem os sintomas, a segunda opção é a disopiramida, que pode aumentar a tolerância ao esforço, às vezes à custa de efeitos colaterais anticolinérgicos, tais como retenção urinária e boca seca.

Quando os betabloqueadores não podem ser utilizados, outra opção é verapamil, embora esse tratamento deva ser cuidadosamente monitorado em pacientes com obstrução severa, devido ao risco de edema pulmonar.

O diltiazem continua sendo a última opção, quando as terapias prévias não forem bem sucedidas.^1^

**Figura 6:**
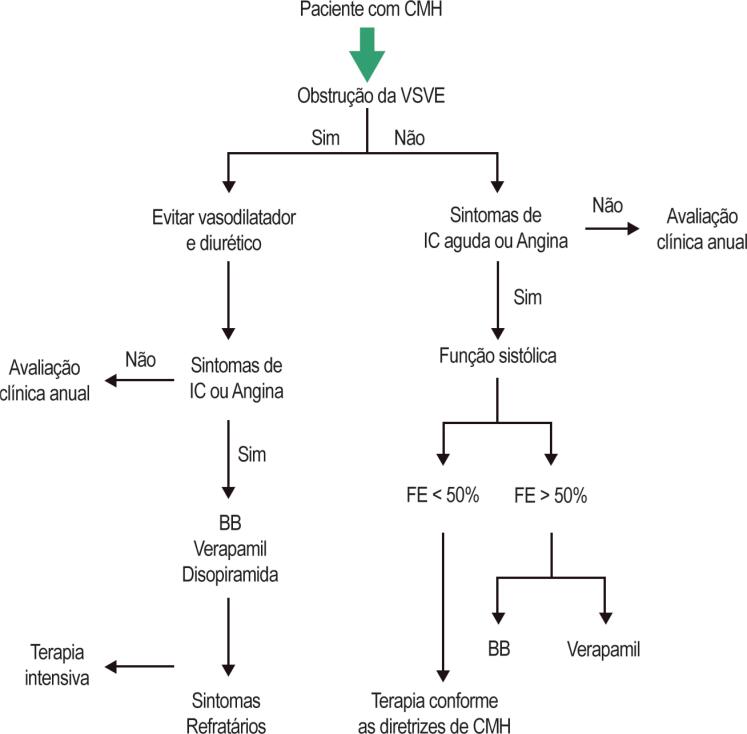
Fluxograma para determinar a medicação apropriada para os pacientes com CMH.

Pacientes que apresentam obstrução da VSVE e sintomas persistentes de IC, apesar da monoterapia, podem se beneficiar da combinação de disopiramida com o tratamento atual implementado^33^ (Figura 6). Os pacientes tratados com disopiramida devem ser submetidos a ECG basal e periódico durante o acompanhamento para monitorar o intervalo QTc. O uso de disopiramida deve ser evitado em pacientes com hiperplasia prostática devido ao seu efeito anticolinérgico.

#### Arritmias e prevenção de morte súbita

A fibrilação atrial (FA) é uma arritmia relativamente comum em pacientes com CMH que potencialmente resulta em desfechos clínicos adversos maiores, e sua incidência é aproximadamente cinco vezes maior em pacientes com CMH quando comparada com a população em geral.^34^

A FA geralmente é pouco tolerada em pacientes com CMH devido à redução do tempo de enchimento diastólico e à perda de contração atrial, fatores que são frequentemente associados à disfunção diastólica, e estão presentes em uma grande proporção desses pacientes. O desenvolvimento de FA está associado a uma piora da classe funcional desses pacientes e aos sintomas da IC.

Além disso, a FA é um marcador de mau prognóstico para pacientes com CMH^35^ e sinaliza um risco significativamente maior de eventos cerebrovasculares agudos.^36^

O tratamento da FA em pacientes com CMH se assemelha às recomendações gerais para tratar a FA em pacientes sem CMH, e tanto o controle do ritmo quanto da frequência cardíaca são opções possíveis.^37^ devendo ser escolhida a melhor estratégia com base no perfil clínico de cada paciente. Uma vez que o risco de eventos tromboembólicos é maior nos pacientes que desenvolvem FA, a recomendação de tratamento anticoagulante nesse grupo de pacientes é aceitável e indicado na maioria dos casos, independentemente da estratificação de risco com base no escore de CHADS2.^27^

As arritmias ventriculares são comuns em pacientes com CMH, incluindo extrassístoles ventriculares (ESV), taquicardia ventricular não sustentada (TVNS), taquicardia ventricular (TV), fibrilação ventricular (FV) e morte cardíaca súbita (MCS). Os primeiros tipos ocorrem com mais frequência em pacientes com CMH.^38^

O tratamento da ESV só é necessário em pacientes que apresentem sintomas, já que a presença desta condição por si só não confere aumento no risco de MCS.^39^

A TVNS ocorre mais frequentemente em pacientes com maiores graus de HVE, em pacientes com classes funcionais mais avançadas (III/IV) e em indivíduos mais velhos. Entretanto, sua presença em indivíduos mais jovens confere um risco maior de MCS. Os episódios de TVNS são mais frequentes durante o sono ou durante outros períodos de hiperatividade vagal. Os pacientes com CMH que apresentam TVNS durante o ECG Holter têm maior risco de MCS^39^ e esse risco é ainda maior se os episódios de TVNS são prolongados, repetitivos, ou associados a sintomas de baixo débito cardíaco.^40^ Quando a terapia farmacológica adjuvante é proposta com o objetivo de reduzir os sintomas ou a incidência de arritmias ventriculares, o medicamento mais comumente utilizado como terapia inicial é o betabloqueador, e a amiodarona tem sido usada para tratar casos refratários.^41^ Nos pacientes com alto risco de desenvolver MCS, nenhuma medicação substitui adequadamente a implantação de um cardioversor-desfibrilador implantável (CDI).

Já foi documentado clinicamente que a TV sustentada é geralmente rara e se manifesta principalmente como palpitações, pré-síncope ou síncope. Na ausência de identificação de um possível fator desencadeante, é considerada um fator de risco importante para MCS. A maioria dos pacientes que desenvolvem esse tipo de arritmia recebe o CDI como prevenção secundária.

A estratificação de risco para MCS deve ser realizada em todos os pacientes com CMH. Os dois primeiros fatores de risco principais para essa condição são parada cardíaca abortada e TV sustentada espontânea prévias.^27^ Os pacientes que sobrevivem a um episódio de FV ou VT têm um risco muito elevado de eventos recorrentes, o que justifica a implantação de um CDI para prevenção secundária nesses pacientes.^42^

Outros fatores de risco maiores para prevenção primária foram identificados, uma vez que a maioria dos pacientes não sobrevive ao primeiro episódio de arritmia ventricular.^43^ e porque pode ser a primeira manifestação da doença em indivíduos assintomáticos.

Oito fatores principais são mais comumente considerados na prevenção primária de MCS:^44^

– História familiar (HF) de CMH relacionada à morte cardíaca súbita^45^ (especialmente se a MCS precoce está presente ou se vários indivíduos da mesma família são atingidos);
– Síncope que não é explicada por outra causa;^46^
– TVNS^38^ (especialmente se estiver associada a sintomas ou se ocorrer em indivíduos jovens);
– Resposta anormal da pressão arterial em pacientes com idade inferior a 40 anos ou em pacientes com história familiar de MCS precoce;^47^
– HVE grave (≥30 mm),^48^ principalmente em pacientes com idade inferior a 30 anos;
– RMC com contraste mostrando realce tardio de gadolínio – fibrose identificada, geralmente superior a 15% da massa do VE;
– Disfunção sistólica com fração de ejeção menor que 50%; e
– Aneurisma apical do ventrículo esquerdo, independentemente do tamanho.^49^

Fatores de risco possíveis incluem a idade do paciente no momento do diagnóstico, um gradiente de pressão maior que 30 mmHg na VSVE, disfunção diastólica, isquemia miocárdica e a presença de genótipos de alto risco, dentre outros (Tabela 1).

**Tabela 1:** Preditores de MCS em pacientes com CMH

Fatores clássicos	Fatores possíveis
MCS abortada	Gradiente elevado (superior a 30 mmHg) na VSVE
IC de MCS	Disfunção diastólica
Síncope inexplicada	Isquemia miocárdica
TVNS ao Holter	Realce cardíaco na RMC
PA anormal ao exercício	Mutação de alto risco
HVE grave (>30 mm)	

Pacientes com dois ou três fatores de risco maiores têm uma taxa anual de MCS abortada de aproximadamente 5%, o que justifica a implantação de CDI nessa população.^50^

Desse modo, a maioria das sociedades e organizações profissionais recomenda que pacientes com CMH que apresentem dois ou mais fatores de risco importantes recebam um CDI para a prevenção primária de MCS^27^, embora estudos mostrem que a presença de um fator de risco maior justifique a implantação de um CDI.^42^^,^^51^

Recentemente, foi desenvolvido um novo modelo para estratificação de risco. Esse escore usa uma equação que introduz variáveis contínuas, tais como idade, fração de encurtamento do ventrículo esquerdo, espessura máxima do ventrículo esquerdo, gradiente máximo VSVE e diâmetro do átrio esquerdo, e demonstrou ser promissor na busca por um método mais preciso para determinar o prognóstico de pacientes com CMH.^52^

### Terapia invasiva

Um gradiente de pressão VSVE ocorre na maioria dos pacientes com CMH,^23^ e representa um pior fator prognóstico e preditor do surgimento de sintomas de IC quando presente em repouso.^24^ Pacientes com obstrução da VSVE e gradiente de pressão no VE/aorta (seja em repouso ou induzida) > 50 mmHg, e que persiste com sintomas limitadores apesar do uso terapia medicamentosa otimizada máxima são candidatos à redução septal invasiva.

A miectomia septal é uma boa opção quando a válvula mitral ou anormalidades do músculo papilar devem ser corrigidas ou a revascularização do miocárdio é necessária, além de remover diretamente o músculo septal e expandir a VSVE.^53^ A miectomia geralmente resulta na resolução do gradiente VSVE e melhora os sintomas dos pacientes,^54^ além de estar associada com excelente sobrevida a longo prazo.^55^

A ablação septal percutânea com álcool é também uma boa alternativa, já que nenhuma meta-análise favoreceu um método até o momento. É particularmente indicada quando a miectomia não deve ser realizada devido ao alto risco cirúrgico ou ao desejo do paciente. Esse procedimento reduz a obstrução da VSVE, promove melhora na classe funcional, e aumenta a capacidade de exercício.^56^ Pacientes submetidos à ablação com álcool apresentam uma taxa de sobrevida de cinco anos, o que é comparável aos pacientes submetidos à miectomia septal e à população em geral.^57^

A principal vantagem da miectomia septal em relação à ablação com álcool é a taxa reduzida de necessidade de implantação de um marcapasso (MP) definitivo devido a um bloqueio atrioventricular avançado, uma necessidade reduzida de reintervenção por causa da recorrência da obstrução VSVE, e uma redução no gradiente VE/aorta após o procedimento.^58^ Além disso, ao contrário da ablação septal, a miectomia septal mostrou reduzir os riscos de MCS e choques inapropriadas do CDI.^59^

A implantação de um MP bicameral programado no modo DDD é uma opção razoável durante a miectomia para reduzir o gradiente VSVE e melhorar os sintomas relacionados a essa condição. Entretanto, essa indicação se restringe a pacientes que já tiveram um dispositivo bicameral por outras indicações, já que não há dados sobre os efeitos a longo prazo da estimulação do ventrículo direito sobre o ventrículo esquerdo com CMH, e o benefício se restringe apenas a um pequeno subconjunto de pacientes.^1^^,^^60^

#### Rastreio familiar

Levando-se em conta a causa genética da CMH, os parentes próximos dos indivíduos afetados devem ser avaliados periodicamente devido à possibilidade de herdar a doença. A avaliação consiste de anamnese, exame físico, ECG, e ecocardiograma como estratégia para detecção precoce da CMH^61^ São recomendadas avaliações a cada 12-18 meses, a partir dos 12 anos de idade, e a cada 5 anos, a partir dos 18 anos.^62^ O ecocardiograma com Doppler tecidual permite detectar alterações na contração e relaxamento ventricular que podem prever o surgimento de disfunção miocárdica nesses pacientes.^63^ No entanto, a presença dessas anomalias não é levada em conta no diagnóstico de CMH.

Os testes genéticos não são realizados rotineiramente no rastreio familiar, exceto em situações nas quais a mutação que causa a CMH tenha sido identificada no caso índice. Nessa situação, a condição genética dos membros da família deve ser determinada. Contudo, a mutação geralmente só é detectada em aproximadamente 35% de todos os pacientes. Por outro lado, se o caso índice tem a mutação e o membro da família não tem, a probabilidade de aparecimento da doença é muito baixa.^4^

#### Profilaxia de endocardite

Os pacientes com CMH apresentam maior risco de desenvolver endocardite infecciosa (EI) quando comparados com pacientes sem CMH, de acordo com um estudo que mostrou um aumento da incidência de EI da valva mitral em pacientes com CMH obstrutiva e com aumento do átrio esquerdo.^64^ Entretanto, revisões recentes das diretrizes internacionais não recomendam a administração rotineira de profilaxia para pacientes com CMH.^1^ Por outro lado, as opiniões dos especialistas continuam favoráveis à manutenção da profilaxia para endocardite nesse grupo de pacientes antes de procedimentos dentários, principalmente em pacientes com CMH obstrutiva.
