# Supplementary material for: Cardiomiopatia Hipertrófica – Revisão
Source: Arq Bras Cardiol. 2020 Nov 1;115(5):927–35. [Article in Portuguese] doi: 10.36660/abc.20190802 (PMC8452207; doi:10.36660/abc.20190802)
Supplement: Translation

# Translation: Hypertrophic Cardiomyopathy: A Review

**Authors:** Silméia Garcia Zanati Bazan, Gilberto Ornellas de Oliveira, Caroline Ferreira da Silva Mazeto Pupo da Silveira, Fabrício Moreira Reis, Karina Nogueira Dias Secco Malagutte, Lucas Santos Nielsen Tinasi, Rodrigo Bazan, João Carlos Hueb, Katashi Okoshi

**Affiliations:** 1 BotucatuSP Brazil Universidade Estadual Paulista Júlio de Mesquita Filho – Faculdade de Medicina de Botucatu - UNESP, Botucatu, SP - Brazil

**Keywords:** Cardiomyopathy, Hypertrophic/genetics, Sudden Cardiac Death, Heart failure, Echocardiography/methods, Hypertrophy, Left Ventricle

## Abstract

Hypertrophic cardiomyopathy (HCM) is the most common heart disease with a genetic origin, and its main characteristic is left ventricular hypertrophy that occurs in the absence of other conditions that trigger this change. HCM may present from asymptomatic forms to manifestations of sudden cardiac death and severe heart failure. Contemporary high-resolution imaging methods and more accurate clinical scores have been used and developed to provide a prognostic assessment and adequate functional assessments, as well as to allow for the stratification of clinical severity. These aspects will be addressed in this review, along with other classic topics inherent to the study of this disease.

## Introduction

Hypertrophic cardiomyopathy (HCM) is a disease with a genetically determined cause that leads to structural changes in the cardiac conformation (Figure 1). The main anatomical characteristic of this disease is left ventricular hypertrophy (LVH) with various morphologies in the absence of other conditions that justify this finding.^1^

**Figure 1:** Schematic of a normal heart (left panel) and a heart with HCM (right panel).

The prevalence of HCM is relatively frequent, occurring in about 0.2% of the adult population.^2^ Its clinical presentation is extremely variable, ranging from asymptomatic forms to advanced heart failure (HF), among other presentations that culminate with sudden death.^3^

On the other hand, advances in the treatment of HCM have resulted in a current mortality rate of less than 1% per year.^4^^,^^5^

Thus, it is a subject of great interest due to its significant prevalence and the importance of early identification of at-risk groups, such as competitive athletes.

### Genetic bases

Genetic analyses of HCM have identified a series of mutations in over 11 genes encoding sarcomeric proteins.^6^ HCM can occur in a dominant autosomal inheritance pattern with variable expressivity and penetrance related to age or as a new mutation in non-family related cases.^7^^,^^8^ The predominant mutation is the missense mutation, in which one nucleic acid is replaced by another, with a subsequent modification of the translated amino acid and the functional property of the resulting protein. Insertions and deletions are also common mutations identified as being involved in the pathogenesis of HCM that trigger the production of abnormal proteins.^8^

Patients with HCM are found to have some type of genetic alteration in approximately one-half cases.^9^^,^^10^

Most mutations affect the genes encoding the contractile proteins of the cardiac sarcomere: troponin T and I myosin light chain, myosin heavy chains alpha and beta, myosin-binding protein C, alpha-actin, alpha-tropomyosin and titin. However, mutations in nonsarcomeric protein-coding genes have already been identified in patients with HCM.^11^ The genes most commonly related to the development of the disease are myosin heavy chain beta (MYH7), myosin-binding protein C (MYBPC3) and troponin T (TNNT2)^12^ (Figure 2).

**Figure 2:** Distribution of gene mutations in HCM (Adapted from Maron BJ et al.^8^)

The pathogenicity of a mutation is evaluated probabilistically using a series of criteria that will determine the risk of developing HCM.^13^

The concept of phenocopies in the HCM context is also important to highlight. These patients have a CHM phenotype without the CMH genetic mutations, but instead present with some other disease leading to a similar heart condition, such as Fabry disease, LAMP2 cardiomyopathy, PRKAG2 and/or amyloidosis.

### Pathological findings

Histopathological analysis of HCM tissue shows hypertrophied myocardial fibers distributed in a disorganized pattern and interposed in a variable amount of interstitial fibrosis^14^ (Figure 3).

**Figure 3:** Myocyte disarray in the myocardial tissue of a patient with HCM.

In addition, intramural coronary arterioles are structurally abnormal and present a decreased intraluminal area with deteriorated vasodilatory capacity, which promotes inefficient blood flow during stress.^15^ Over time, repeated episodes of ischemia lead to cell death, and repair is mediated by replacement with fibrotic tissue.^14^

Different types of anatomical presentations of HCM have been reported. The most common type is asymmetric septal hypertrophy (present in > 75% of cases), followed by apical, concentric, medioventricular and lateral presentations.^16^

### Pathophysiology

HCM-related symptoms are related to the combination of diastolic dysfunction, obstruction of the left ventricular outflow tract (LVOT), mitral regurgitation, myocardial ischemia and arrhythmias. The most common factor contributing to the development of LVOT obstruction is systolic anterior motion (SAM) of the mitral valve against the IVS. SAM occurs due to the high speed of blood flow through the LVOT that drags the anterior mitral valve leaflet toward the interventricular septum, resulting in a direct impediment to blood flow through the outflow tract.^17^

In addition, the combination of myocyte disarray, autonomic disorder, LVH, ischemia and myocardial fibrosis generates a sufficient arrhythmogenic substrate for the development of the main arrhythmias observed in patients with HCM.^2^

These characteristics do not appear simultaneously, and a 4-stage classification has been proposed to assist with the diagnosis and management of patients: nonhypertrophic HCM, classic phenotype, adverse remodeling and overt dysfunction.^18^ As the patient advances through the stages, he experiences a loss of ejection fraction, an increase in the left ventricular mass, a worsening of microvascular and diastolic dysfunction, an intensification of symptoms and a loss of a prior left ventricular outflow tract obstruction, which usually begins in stage 2.

### Clinical presentations

HCM-related symptoms are related to the existing profiles of the disease, including an asymptomatic presentation, sudden cardiac death-ventricular arrhythmias, obstruction, heart failure with preserved ejection fraction, atrial fibrillation/stroke and heart failure with reduced ejection fraction. Although many patients with HCM have no symptoms or only experience minor symptoms, others may present dyspnea under stress, fatigue, chest pain, pre-syncope and syncope, during or shortly after stress, and heart palpitations.^19^

A well-established correlation has been observed between the presence or magnitude of the LVOT obstruction and the presence of symptoms.^20^

For most patients with HCM, LVH is not progressive and is compatible with a normal lifespan, with an annual mortality rate of approximately 1%.^21^

On the other hand, a small group of patients present a risk of developing symptoms related to the progression of systolic heart failure, sudden death, and atrial fibrillation related to thromboembolic phenomena.^22^

The presence of a pressure gradient in the LVOT at rest or provoked by exercise occurs in most patients with HCM.^23^ Significant obstruction at rest is an independent factor for a worse prognosis and progression to heart failure.^24^

The physical examination of patients with HCM may reveal normal findings or the presence of various signs, such as the fourth heart sound (S4), regurgitation systolic heart murmur on the lower left sternal border, paradoxical splitting of second heart sound (S2), heaving apical impulse, and systolic thrill. Additionally, patients with obstruction of the LVOT may present an ejection systolic murmur at the left sternal edge that usually radiates to the right upper sternal edge and may increase upon standing from the squatting position and in the Valsalva maneuver.

The arterial pulse may be bifid and present a dome-shaped systolic peak, while a prominent “a” wave is detected in the venous pulse.

### Complementary examinations

– Electrocardiogram (ECG): This test should be performed in all patients with suspected HCM. A normal ECG is unusual, as it was observed in less than 10% of patients with HCM, and this test is very sensitive for identifying the disease.^25^ This group of patients tends to present a better prognosis than patients who present electrocardiographic alterations.^26^ The most common abnormal pattern is the presence of localized or diffuse alterations in ventricular repolarization. Other findings may include signs of left ventricular hypertrophy, the inversion of T wave at the left leads, and an increase in the left atrium. Deep and narrow “Q” waves may occur in V5 and V6.
– Echocardiogram: An echocardiogram is an essential examination both for diagnostic confirmation and for evolutionary, functional and prognostic evaluations.^27^ The transthoracic echocardiogram can show the heart morphology, estimate the systolic and diastolic function, assess the presence and severity of the gradient in the LVOT, and determine the degree of mitral regurgitation. The major echocardiographic findings associated with HCM are LVH (particularly if it is asymmetrical and involving the anterolateral wall or septum), an increased gradient in the LVOT, and the systolic anterior motion of the mitral leaflet (Figure 4).

**Figure 4:** Transthoracic echocardiogram showing asymmetrical hypertrophy of the interventricular septum. IVS: interventricular septum; LV: left ventricle; LA: left atrium; AO: aortic root. (Serviço de Ecocardiografia do HC – Faculdade de Medicina de Botucatu – UNESP).

Patients who remain symptomatic and do not present an obstruction at rest may undergo stress echocardiography to induce a gradient and subsequently adjust the therapeutic management and treatment according to the result.^23^

– Holter-ECG: This test is conducted as part of the stratification of the risk of developing ventricular arrhythmias and sudden death, as well as to investigate palpitations and in patients with suspected atrial fibrillation.
– Exercise stress test: This test is typically performed for risk stratification by measuring the blood pressure response to exercise and to investigate ischemia and arrhythmias.
– Cardiac Magnetic Resonance (CMR): CMR provides high-resolution images for evaluating cardiac structures. In addition to being able to identify hypertrophy in segments that are not displayed in echocardiography, it also shows myocardial fibrosis areas, which are usually detected through late gadolinium enhancement, and are one of the sudden death risk factors, enabling better characterization of structural abnormalities in the mitral valve apparatus^28^^–^^30^ (Figure 5).

**Figure 5:** CMR of a patient with HCM and the nonobstructive asymmetric septal presentation. LA: left atrium; RA: right atrium; IVS: interventricular septum; LV: left ventricle; RV: right ventricle; LVOT: left ventricular outflow tract. (Courtesy of the Department of Radiology of the HC – Faculdade de Medicina de Botucatu – UNESP).

### Treatment

The treatment is initiated with preventive measures, such as avoiding intravascular volume depletion and restricting the practice of intense physical exercise, with the recommended activity level being individualized for each patient.^31^^,^^32^ Additional measures include the maintenance of negative inotropic drugs, avoiding the use of vasodilators and the use of an appropriate treatment for tachyarrhythmias.

#### Drug therapy

Pharmacological therapy is the first-line treatment for patients with symptoms of HF related to an LVOT obstruction.^26^

The use of medications is not recommended before the development of symptoms, since evidence does not indicate that pharmacological therapy changes the natural history of asymptomatic patients.

The first-line treatment is beta-blockers. Currently, clinical trials have not indicated preference for a specific beta-blocker, as they have not been compared. However, studies have reported the benefits of propranolol and sotalol, although the latter is a class 3 anti-arrhythmic agent, in reducing the symptoms and decreasing arrhythmias.

Upon the failure of beta-blockers to alleviate the symptoms, the second option is disopyramide, which may increase effort tolerance, sometimes at the cost of anticholinergic side effects, such as urinary retention and dry mouth.

When beta-blockers are unable to be used, another option is verapamil, although this treatment must be carefully monitored in patients with severe obstruction due to the risk of pulmonary edema.

Diltiazem remains the last option when the previous therapies were unsuccessful.^1^

Patients who present an LVOT obstruction and persistent symptoms of HF despite monotherapy may benefit from the combination of disopyramide with the current treatment implemented^33^ (Figure 6). Patients treated with disopyramide should undergo basal and periodic ECG during follow-up to monitor the QTc interval. The use of disopyramide should be avoided in patients with prostatic hyperplasia due to its anticholinergic effect.

**Figure 6:** Flow chart to determine the appropriate medication for patients with HCM.

#### Arrhythmias and prevention of sudden death

Atrial fibrillation (AF) is a relatively common arrhythmia in patients with HCM that potentially results in major adverse clinical outcomes, and its incidence is approximately five times greater in patients with HCM than in the general population.^34^

AF is usually poorly tolerated in patients with HCM due to the reduction in diastolic filling time and loss of atrial contraction, factors that are often associated with diastolic dysfunction and are present in a large proportion of these patients. The development of AF is associated with a worsening of the functional class of these patients and with symptoms of HF.

In addition, AF is a marker of poor prognosis for patients with HCM^35^ and signals a significantly increased risk of acute cerebrovascular events.^36^

The treatment of AF in patients with HCM is similar to the general recommendations for treating AF in patients without HCM, and both the control of rhythm and heart rate are available options,^37^ with the choice of the best strategy being based on the clinical profile of each patient. Since the risk of thromboembolic events is increased in patients who develop AF, the recommendation of anticoagulant treatment in this group of patients is reasonable and indicated in most cases, regardless of the risk stratification based on the CHADS2 score.^27^

Ventricular arrhythmias are common in patients with HCM, including ventricular extrasystoles (VES), non-sustained ventricular tachycardia (NSVT), ventricular tachycardia (VT), ventricular fibrillation (VF), and sudden cardiac death (SCD). The first two types occur more frequently in patients with HCM.^38^

The treatment of VES is only necessary in patients who present symptoms, since the presence of this condition alone does not confer an increased risk of SCD.^39^

NSVT occurs more frequently in patients with higher degrees of LVH, in patients with more advanced functional classes (III/IV) and in older individuals. However, its presence in young individuals confers a greater risk of SCD. NSVT episodes are more frequent during sleep or during other periods of vagal hyperactivity. Patients with HCM who present NSVT during a Holter-ECG exhibit an increased risk of SCD,^39^ and this risk is even higher if the episodes of NSVT are prolonged, repetitive, or associated with symptoms of low cardiac output.^40^ When adjuvant pharmacological therapy is proposed to reduce symptoms or the incidence of ventricular arrhythmias, the medicine that is most commonly used as the initial therapy is the beta-blocker, and amiodarone has been used to treat refractory cases.^41^ In patients at high risk of developing SCD, no drug is a suitable alternative to the implantation of an implantable cardioverter-defibrillator (ICD).

Clinically documented, sustained VT is usually rare and presents mostly as palpitations, pre-syncope or syncope. In the absence of the identification of a possible triggering factor, it is considered a major risk factor for SCD. Most patients who develop this type of arrhythmia receive the ICD as a secondary prevention strategy.

Risk stratification for SCD should be performed in all patients with HCM. The first two major risk factors for this condition are prior aborted cardiac arrest and spontaneous sustained VT.^27^ Patients who survive an episode of VF or VT are at very high risk of recurring events, which justifies the implant of an ICD for secondary prevention in these patients.^42^

Additional major risk factors for primary prevention have been identified, since the majority of patients do not survive the first episode of ventricular arrhythmia,^43^ and because it may be the first manifestation of the disease in asymptomatic individuals.

Eight major factors are more commonly considered in the primary prevention of SCD:^44^

– A family history (FH) of HCM related to sudden cardiac death^45^ (particularly if early SCD is present or multiple individuals within the same family are affected);
– Syncope that is not explained by another cause;^46^
– NSVT^38^ (particularly if it is associated with symptoms or occurs in young individuals);
– Abnormal response of blood pressure in patients aged less than 40 years or patients with a family history of early SCD;^47^
– Severe LVH (≥30 mm),^48^ particularly in patients aged less than 30 years;
– Contrast CMR showing late gadolinium enhancement - identified fibrosis, usually greater than 15% of the LV mass;
– Systolic dysfunction with an ejection fraction less than 50%; and
– Left ventricular apical aneurysm, regardless of size.^49^

Possible risk factors include the patient's age at the time of diagnosis, a pressure gradient greater than 30 mmHg in the LVOT, diastolic dysfunction, myocardial ischemia and the presence of high-risk genotypes, among others (Table 1).

**Table 1:** Predictors of SCD in patients with HCM

Classical Factors	Possible factors
Aborted SCD	Elevated gradient (above 30 mmHg) in LVOT
HF of SCD	Diastolic dysfunction
Unexplained syncope	Myocardial ischemia
NSVT to Holter	Late enhancement in MRI
BP abnormal to exercise	High-risk mutation
Severe LVH (>30 mm)	

Patients with two or three major risk factors have an aborted SCD rate of approximately 5% per year, which justifies the implantation of an ICD in this population.^50^

Thus, most professional societies and organizations recommend that patients with HCM presenting with two or more major risk factors receive an ICD for the primary prevention of SCD^27^, although studies have shown that the presence of a major risk factor justifies the implantation of an ICD.^42^^,^^51^

Recently, a new model for risk stratification has been developed. This score uses an equation that introduces continuous variables such as age, left ventricular shortening fraction, left ventricular maximum thickness, maximum gradient in the LVOT, and left atrial diameter, and proved to be promising in the search for a more accurate method to determine the prognosis of patients with HCM.^52^

### Invasive therapy

A pressure gradient in the LVOT occurs in most patients with HCM,^23^ and represents a poor prognostic factor and predictor of the emergence of HF symptoms when present at rest.^24^ Patients with an LVOT obstruction and LV/aorta pressure gradient (either at rest or induced) > 50 mmHg and that persist with limiting symptoms despite the use of the maximum optimized drug therapy are candidates for invasive septal reduction.

Septal myectomy is a good option when the mitral valve or papillary muscle abnormalities must be repaired or myocardial revascularization is required, in addition to directly removing the septal muscle and expanding the LVOT.^53^ Myectomy generally results in the resolution of the gradient in the LVOT and improves the symptoms of patients,^54^ in addition to being associated with excellent long-term survival.^55^

Percutaneous alcohol septal ablation is also a good alternative, as no meta-analysis has favored one method to date. It is particularly indicated when myectomy should not be conducted due to a high surgical risk or the desire of the patient. This procedure reduces the LVOT obstruction, promotes improvement in the functional class, and increases exercise capacity.^56^ Patients subjected to alcohol ablation present a five-year survival rate that is comparable to patients subjected to septal myectomy and to the general population.^57^

The main advantage of septal myectomy compared with alcohol ablation are: reduced need for implantation of a definitive pacemaker (PM) due an advanced atrial-ventricular block, reduced need for reintervention because of the recurrence of the LVOT obstruction, and reduced LV/aorta gradient after the procedure.^58^ In addition, in contrast to septal ablation, septal myectomy has been shown to reduce the risks of SCD and inappropriate discharges of the ICD.^59^

The implantation of a DDD bicameral PM is a reasonable option during myectomy to reduce the gradient in the LVOT and improve symptoms related to this condition. However, this indication is restricted to patients who already have a bicameral device for other indications, since data on the long-term effects of right ventricle pacing on an HCM left ventricle are unavailable, and the benefit is restricted to only a small subset of patients.^1^^,^^60^

#### Family screening

Considering the genetic cause of HCM, close relatives of affected individuals should be evaluated periodically due to the possibility of inheriting the disease. The evaluation consists of anamnesis, physical examination, ECG and echocardiogram, as a strategy for early detection of HCM.^61^ Evaluations every 12-18 months are recommended, starting at the age of 12, and every 5 years beginning at the age of 18.^62^ Echocardiogram with tissue Doppler has been shown to detect alterations in ventricular contraction and relaxation that may predict the emergence of myocardial dysfunction in these patients.^63^ However, the presence of these abnormalities is not considered in the diagnosis of HCM.

Genetic tests are not routinely performed in family screening, except in situations where the mutation causing the HCM has been identified in the index case. In this situation, the genetic status of the family members should be determined. However, the mutation is generally only detected in approximately 35% of all patients. On the other hand, if the index case has the mutation and the family member does not, the likelihood of disease onset is very low.^4^

#### Endocarditis prophylaxis

Patients with HCM present a higher risk of developing infective endocarditis (IE) than patients without HCM, according to a study showing an increased incidence of mitral valve IE in patients with obstructive HCM and with increased size of the left atrium.^64^ However, recent reviews of international guidelines do not recommend the routine administration of prophylaxis for patients with HCM.^1^ On the other hand, specialists' opinions are still in favor of maintaining the prophylaxis for endocarditis in this group of patients before dental procedures, particularly in patients with obstructive HCM.
